# Parliament and the Professions

**Published:** 1920-12-25

**Authors:** 


### Parliament and the Professions.

### Exports of Opium, Morphia, and Cocaine.

Sir P. Lloyd-Graeme informed Mr. Gilbert that exports of opium, morphia, and cocaine and their preparations to United States of America, France, Canada, and Japan are only licensed on production of a certificate signed on behalf of the Government concerned that the consignment is required exclusively for legitimate scientific or medical purposes, and will not be re-exported. The extension to other countries of a similar system of certificates is at present the subject of negotiations.

### Smallpox in Scotland.

The Secretary for Scotland stated that the outbreak of smallpox in Scotland this year began in Glasgow at the end of February. Cases have since occurred in the areas of thirty-three other local authorities in Scotland. Up to the 8th inst., of 516 cases in Glasgow 107 were fatal. Of the cases the number previously vaccinated was 394, of whom 61 died; the number unvaccinated was 115, of whom 43 died. Outside Glasgow there were 180 cases, of whom 137 were previously vaccinated, 42 unvaccinated.

### The Prophylactic Value of Whisky.

Mr. MacQuisten asked the Minister of Health whether, seeing that whisky and aspirin are in combination unfailing prophylactics for severe colds and influenza, and that suffering and deaths have been and are being caused to those who are unwilling or unable to buy whisky in bulk, but would purchase and use it medicinally, he will take steps to have the Regulation anent the sale in homœopathic quantities annulled. Dr. Addison replied that he is advised that the prophylactic value of whisky, whether in conjunction with aspirin or otherwise, in the treatment of influenza cannot be regarded as sufficiently established to justify the adoption of the suggestion.

### Danish Hospitals.

Mr. Swan asked the Minister of Health whether the hospitals and infirmaries of Denmark are State-owned and controlled; whether any charge is demanded from those who need treatment, and whether such services are efficient. Dr. Addison replied that there is one State hospital—at Copenhagen—and a number of voluntary hospitals provided by religious and philanthropic bodies; but the majority of general hospitals are provided and controlled by the local authorities. Payment is made for all patients, either by the patient himself, by the sickness society of which he is a member, or by the Poor-Law authority. The Minister said that information available is not sufficient to enable him to express any opinion as to the efficiency of the hospital services.

